# Suppressor of fused-restrained Hedgehog signaling in chondrocytes is critical for epiphyseal growth plate maintenance and limb elongation in juvenile mice

**DOI:** 10.3389/fcell.2022.997838

**Published:** 2022-09-02

**Authors:** Chunmei Xiu, Tingting Gong, Na Luo, Linghui Ma, Lei Zhang, Jianquan Chen

**Affiliations:** ^1^ Orthopedic Institute, Suzhou Medical College of Soochow University, Suzhou, Jiangsu, China; ^2^ Key Laboratory of Novel Targets and Drug Study for Neural Repair of Zhejiang Province, Department of Clinical Medicine, School of Medicine, Zhejiang University City College, Hangzhou, Zhejiang, China; ^3^ Department of Orthopaedics, First Affiliated Hospital of Soochow University, Orthopedic Institute, Suzhou Medical College of Soochow University, Suzhou, Jiangsu, China

**Keywords:** hedgehog signaling, epiphyseal growth plate, skeletal stem cell, suppressor of fused, chondrocyte proliferation and hypertrophy

## Abstract

Hedgehog (Hh) signaling plays multiple critical roles in regulating chondrocyte proliferation and differentiation during epiphyseal cartilage development. However, it is still unclear whether Hh signaling in chondrocytes is required for growth plate maintenance during juvenile growth, and whether sustained activation of Hh signaling in chondrocytes promotes limb elongation. In this study, we first utilized Hh reporter mice to reveal that Hh signaling was activated in resting and columnar chondrocytes in growth plates of juvenile and adult mice. Next, we genetically modulated Hh signaling by conditionally deleting *Smo* or *Sufu* in all or a subpopulation of growth plate chondrocytes, and found that ablation of either *Smo* or *Sufu* in chondrocytes of juvenile mice caused premature closure of growth plates and shorter limbs, whereas *Osx-Cre*-mediated deletion of either of these two genes in prehypertrophic chondrocytes did not lead to obvious growth plate defects, indicating that Hh signaling mainly functions in resting and/or columnar chondrocytes to maintain growth plates at the juvenile stage. At the cellular level, we found that chondrocyte-specific ablation of *Smo* or *Sufu* accelerated or suppressed chondrocyte hypertrophy, respectively, whereas both decreased chondrocyte proliferation and survival. Thus, our study provided the first genetic evidence to establish the essential cell-autonomous roles for tightly-regulated Hh signaling in epiphyseal growth plate maintenance and limb elongation during juvenile growth.

## Introduction

Longitudinal skeletal growth in children is primarily orchestrated by activity of growth plates, the specialized epiphyseal cartilages sandwiched between primary spongiosa and secondary ossification center (Hallett et al., 2019; Chagin and Newton, 2020; Matsushita et al., 2020; Koyama et al., 2021). Similar to other epiphyseal cartilages, the juvenile growth plates are mainly composed of three histologically distinct layers of chondrocytes, including the top resting zone containing the progenitor/stem cells, the middle proliferating zone with flat column-forming chondrocytes, and the bottom hypertrophic zone containing terminally differentiated chondrocytes that could either undergo apoptosis or convert into osteoblasts directly or via marrow-associated skeletal stem and progenitor cells (Yang et al., 2014; Aghajanian and Mohan, 2018; Hallett et al., 2019; Long et al., 2022). However, unlike other epiphyseal cartilages, formation of juvenile growth plates is accompanied by maturation of the secondary ossification center (SOC), which could influence growth plate growth and maintenance. Indeed, bony epiphyses in the SOC were recently shown to function as a niche that generated and maintained self-renewing growth plate skeletal stem cells (gpSSCs) within the resting zone (Mizuhashi et al., 2018; Newton et al., 2019; Chagin and Newton, 2020; Kurenkova et al., 2020). Accordingly, chondrocytes in juvenile growth plates are produced from asymmetric division of gpSSCs, rather than consumption of chondroprogenitors observed in fetal/neonatal mice (Mizuhashi et al., 2018; Newton et al., 2019; Chagin and Newton, 2020). Moreover, this unique SOC niche appears to alter behaviors of growth plate chondrocytes, since growth plate chondrocytes exhibit differential responses to modulators of Hedgehog signaling before and after onset of SOC formation (Mizuhashi et al., 2018; Newton et al., 2019; Kurenkova et al., 2020). Despite these differences, our current understanding of the mechanism regulating chondrocyte proliferation and differentiation is mainly derived from genetic studies of cartilage development at fetal and neonate stages before the onset of SOC formation (Kurenkova et al., 2020; Ohba, 2020). Whether and how these mechanisms are involved in growth plate maintenance during juvenile growth remain to be specifically tested.

Indian hedgehog (Ihh) signaling plays multiple critical roles in the regulation of chondrocyte proliferation and differentiation during epiphyseal cartilage development at both embryonic and neonatal stages (St-Jacques et al., 1999; Long et al., 2001; Razzaque et al., 2005; Maeda et al., 2007; Amano et al., 2015; Ohba, 2020). Interestingly, Ihh continues to be expressed in prehypertrophic chondrocytes of the growth plates and some osteoblast-like cells at the chondro-osseous junction in juvenile mice (Maeda et al., 2007; Zhang et al., 2022). In addition, Sonic hedgehog (Shh) molecules are expressed by several types of cells residing in the SOC (Newton et al., 2019). These two Hh ligands might activate Hh signaling in the growth plate chondrocytes of juvenile mice. In agreement with this prediction, activity of Hh signaling was indeed detected in the growth plate chondrocytes in juvenile and adult mice (Shi et al., 2017; Haraguchi et al., 2018; Newton et al., 2019; Zhang et al., 2022), implying a direct requirement of Hh signaling in chondrocytes. Consistently, pharmacological inhibition of Hh signaling during or after SOC formation led to premature closure of long bone growth plates (Kimura et al., 2008), confirming the functional significance of Hh signaling in epiphyseal growth plate maintenance during juvenile growth. However, different treatment regimens appeared to exert differential cellular effects on chondrocytes or their progenitors. Treatment of mice with *Smo* antagonists HhAntag for 5 days from postnatal (P) 10–14 days (P10-14) or LDE225 (sonidegib) for 2 days (P22-P23) both led to impaired columnar chondrocyte proliferation and accelerated chondrocyte hypertrophy (Kimura et al., 2008; Koyama et al., 2021). In contrast, 6 administrations of *Smo* antagonist vismodegib at P31-P34 did not affect columnar chondrocyte proliferation and hypertrophy (Newton et al., 2019). Similarly, mice subjected to 2 doses of LDE225 (sonidegib) exhibited a decrease in the number of reserve progenitors (Koyama et al., 2021), whereas pharmacological inhibition or activation of Hh signaling (*via* 6 administrations of vismodegib or *Smo* agonist SAG at P31-P34, respectively) did not alter the number of CD73^+^ epiphyseal stem cells within the resting zone, but instead affect their cycling (Newton et al., 2019). Thus, while the above pharmacological studies have established the essential role of Hh signaling in juvenile growth plates, the underlying cellular mechanisms are still unclear. Furthermore, since chondrocyte-specific ablation of *Smo*, the gene encoding the G protein-coupled receptor Smoothened *(Smo)* that is essential for cells responding to Hh signals, caused early lethality and severe defects in endochondral bone development (Long et al., 2001), while genetic deletion of *Smo* in hypertrophic chondrocytes did not result in notable growth plate defects in juvenile mice (Wang et al., 2022). Therefore, it remains to be determined whether the effects of Hh molecules on growth plates is mediated by the direct activation of Hh signaling in chondrocytes.

Hh signaling is tightly regulated by several key regulators, one of which is suppressor of fused *(Sufu)* (Wu et al., 2017; Ohba, 2020). *Sufu* inhibits production and transcriptional activity of full-length Gli transcriptional activators, while facilitating formation of truncated Gli transcriptional repressors (Jiwani et al., 2020). Thus, through reducing Gli activator to repressor ratio, *Sufu* restrains excessive Hh signaling, whereas inactivation of *Sufu* leads to constitutively activated Hh signaling. Intriguingly, *Sufu* could exert either inhibitory or stimulatory effect on cell proliferation and differentiation in different tissues, suggesting that cellular effects of *Sufu*-mediated restraint of Hh signaling is highly context-dependent (Hsu et al., 2011; Li et al., 2017; Noguchi et al., 2019; Yung et al., 2019; Feng et al., 2020; Jiwani et al., 2020). During embryonic epiphyseal cartilage development, *Sufu* promotes chondrocyte proliferation, while inhibiting chondrocyte hypertrophy (Hsu et al., 2011). However, its role in growth plate chondrocytes during juvenile growth is unclear.

In this study, we explored the role of chondrocyte-specific Hh signaling in growth plate maintenance during juvenile growth. By utilizing Hh reporter mice, we revealed that Hh signaling is activated in resting and columnar chondrocytes in the epiphyseal growth plates of juvenile and adult mice. By genetically modulating Hh signaling in all or a subpopulation of growth plate chondrocytes, we demonstrated that Hh signaling mainly functions in immature chondrocytes to regulate chondrocyte turnover and hypertrophy during juvenile life in mice, and that *Sufu*-mediated restraint of Hh activity is critical for maintaining juvenile growth plates.

## Materials and methods

### Mice

*Sufu* conditional knockout mice (*Sufu* ^*f/f*^) (Li et al., 2015) were generated and kindly provided by Dr. Zunyi Zhang (Hangzhou Normal University, China). *Gli1-LacZ* mice, *Ptc1-LacZ*, *Smo* conditional knockout mice (*Smo* ^*f/f*^), *Agc1-CreER* ^*T2*^, and *Osx-Cre* mice were described previously (Goodrich et al., 1997; Long et al., 2001; Bai et al., 2002; Rodda and McMahon, 2006; Henry et al., 2009). All animal used in this study were maintained in a standard specific pathogen-free (SPF) barrier facility at Laboratory Animal Center of Soochow University. All animal experiments were performed following the protocol approved by the Ethics Committee of Soochow University.

### Tamoxifen injection

Tamoxifen (T5648, Sigma) was dissolved in corn oil (C8267, Sigma) at a concentration of 10 mg/ml and stored in dark place. To specifically ablate *Smo* or *Sufu* in growth plate chondrocytes of juvenile mice, 2-week-old *Agc1-CreER* ^*T2*^ *; Smo* ^*fl/fl*^ mice (*Smo* ^*Agc1*^), *Agc1-CreER* ^*T2*^ *; Sufu* ^*fl/fl*^ (*Sufu* ^*Agc1*^) mice or their respective littermate controls were injected intraperitoneally with 100 mg/kg body weight of tamoxifen once daily for 5 consecutive days.

### Micro-computed ttomography analysis

Hindlimbs were dissected from mice and fixed with 10% neutral buffered formalin (Sangon Biotech, China) for 48 h. After being thoroughly washed with PBS (pH7.4), samples were subjected to μCT scanning as previously described (Sun et al., 2022). Measurement of tibia or femur length was performed on coronal images using DataViewer software.

### X-gal/LacZ staining of tibial sections

X-gal/LacZ staining was performed to monitor β-galactosidase (β-gal, a product of the *LacZ* gene) activity as previously described (Han and Feng, 2014). Briefly, tibiae from mice carrying *Gli1-LacZ* or *Ptc1-LacZ* allele were fixed with 1% PFA at 4°C overnight and decalcified with 0.1 M EDTA solution at 37°C for 3 d with gentle agitation. Subsequently, samples were processed for O.C.T. embedding and cryosectioned at 6 μm thickness. Sections were washed three times with LacZ washing buffer (PBS containing 0.02% Nonidet P-40, 0.01% sodium deoxycholate, and 2 mM MgCl_2_), followed by incubation with LacZ staining buffer (LacZ washing buffer supplemented with 5 mM potassium ferricyanide, 5 mM potassium ferrocyanide, and 0.5 mg/ml X-gal) at 37°C overnight. Afterwards, sections were counterstained with nuclear fast red solution, dehydrated in 75 and 100% ethanol, cleared in xylenes, and then mounted with neutral resin.

### Histological analyses

Paraffin or frozen sections of long bones were prepared as we previously described (Zhang et al., 2022). Hematoxylin and eosin (H&E) staining was performed following the standard protocol. For Safranin O/Fast green staining, sections were first stained with hematoxylin for 2 min, followed by differentiation in 1% acid alcohol. Subsequently, the samples were stained with 0.02% Fast Green (F7252, Sigma) for 2 min, rinsed with 1% acetic acid for 30 s, and then stained with 1% Safranin O (S8884, Sigma) for 30 min. Next, slides were dehydrated in graded ethanol series, cleared in xylene, applied with one drop of neutral balsam mounting medium, and finally covered with a coverslip. Images were acquired using a Zeiss Axio Imager Z2 upright microscope (Carl Zeiss Microscopy).

### TUNEL staining

To detect the apoptotic cells, TUNEL (terminal deoxynucleotidyl transferase dUTP nick end labeling) staining was performed on paraffin sections of long bones using a commercial kit (*In Situ* Cell Death Detection Kit, TMR Red; Roche, Mannheim, Germany) according to the manufacturer’ protocol. Briefly, tibial sections were de-paraffinized, rehydrated, and then permeabilized with 0.1% Triton X-100 in 0.1% sodium citrate at room temperature for 8 min. Afterwards, slides were incubated with TUNEL reaction mixture (9:1 mixture of label solution and enzyme solution provided by the kit) at 37°C for 1 h. After washing twice with phosphate buffer solution (PBS), samples were counter-stained with DAPI (C1005, Beyotime Biotechnology, Shanghai, China). Finally, slides were mounted with anti-fade mounting medium and analyzed under a fluorescence microscope.

### BrdU labeling and staining

For BrdU (5-bromo-2′-deoxyuridine) labelling, mice were intraperitoneally injected with 10 μl per gram body weight of BrdU labeling reagent (#000103, Invitrogen) 6 h before being sacrificed. The hindlimbs were then harvested and processed for paraffin sections. To detect BrdU-labeled proliferating cells, tibial sections were dewaxed, rehydrated, and then denatured with 2 N HCl, prior to antigen retrieval with 0.125% trypsin in PBS at 37°C for 10 min. After blocking with 10% goat serum for 1 h, sections were incubated with rat anti-BrdU antibody (ab6326, Abcam, 1:100) at 4°C overnight, followed by incubation with Alexa Fluor 647-conjugated donkey anti-rat (ab150155, Abcam,1:200) or Alexa Fluor 488-conjugated goat anti-rat secondary antibody (ab150157, Abcam, 1:200). Finally, nuclei were counterstained with DAPI (C1005, Beyotime Biotechnology, Shanghai, China) to stain the cell nuclei, and subsequently mounted with anti-fade mounting medium.

### Alkaline phosphatase staining

To assess ALP activity, ALP staining was performed on frozen sections of tibias. Briefly, tibial sections were washed three times with PBS, and then incubated with 0.1 M Tris-HCl solution (pH8.5) containing 0.1 mg/ml naphthol AS-MX phosphate, 0.5% N, N-dimethylformamide, 2 mM MgCl_2_, and 0.6 mg/ml of fast blue BB salt for 20–60 min. Next, sections were dehydrated in graded ethanol series, cleared in xylene, applied with one drop of neutral balsam mounting medium, and finally covered with a coverslip. Images were acquired using a Zeiss Axio Imager Z2 upright microscope (Carl Zeiss Microscopy).

### Immunofluorescence and immunohistochemistry staining

IF was performed on frozen sections. To unmask antigen epitopes for Collagen II, Collagen X, and Mmp13 antibodies, section samples were pretreated with 2 mg/ml hyaluronidase (Sigma, H3506) at 58°C for 2 h. After antigen retrieval, sections were incubated with 10% goat serum in PBS to block nonspecific binding, followed by incubation with primary antibodies in a humidified chamber at 4°C overnight. On the next day, Cy3-conjugated goat anti-rabbit secondary antibody (GB21303, Servicebio, 1:400) were applied to detect primary antibodies, and then DAPI was used to stain cell nuclei.

IHC was carried out on paraffin sections following the standard protocol. Briefly, paraffin sections were deparaffinized, rehydrated, and then subjected to antigen retrieval as described above. Subsequently, 3% hydrogen peroxide in methanol was used to quench endogenous peroxidase activity, prior to blocking nonspecific antigens with 10% goat serum. Slides were then incubated with properly diluted primary antibodies in a humidified chamber at 4°C overnight, followed by incubation with HRP-conjugated goat anti-rabbit secondary antibody (D110058-0025, BBI Life Science, 1:400). For visualizing antibody binding, sections were covered with 3,3′-diaminobenzidine (DAB) freshly-prepared from a DAB substrate kit (ZLI-9018, ZSGB-BIO, Beijing, China) until brown color developed. The stained sections were counterstained with hematoxylin solution, dehydrated in ethanol, cleared in xylene, and finally sealed with a coverslip.

Primary antibody against Collagen II was purchased from Fitzgerald (70R, 1:200). Primary antibodies against Collagen X (ab58632, 1:750) and Mmp13 (ab39012, 1:200) were both obtained from Abcam. Images were acquired using a Zeiss Axio Imager Z2 upright microscope (Carl Zeiss Microscopy).

### Statistical analysis

Data plotting and statistical analyses were performed using GraphPad Prism 8 (GraphPad Software, San Diego, CA). Data were plotted as dot-plots showing individual data points together with mean ± standard deviation. Each data dot represents the value from one mouse. Comparison of means between two groups and among multiple groups were assessed by Student’s t-test and by one-way ANOVA with Tukey’s post-hoc test, respectively. In both cases, a *p* value <0.05 was considered to be statistically significant, and degrees of statistical significance were further designated by different numbers of asterisk (*) in the figures: *, *p < 0.05*; **, *p < 0.01*; ***, *p < 0.001*; ****, *p < 0.0001*.

## Results

### Hh signaling is active in columnar and resting chondrocytes, but downregulated in hypertrophic chondrocytes, in the epiphyseal growth plates of juvenile and adult mice

**FIGURE 1:**
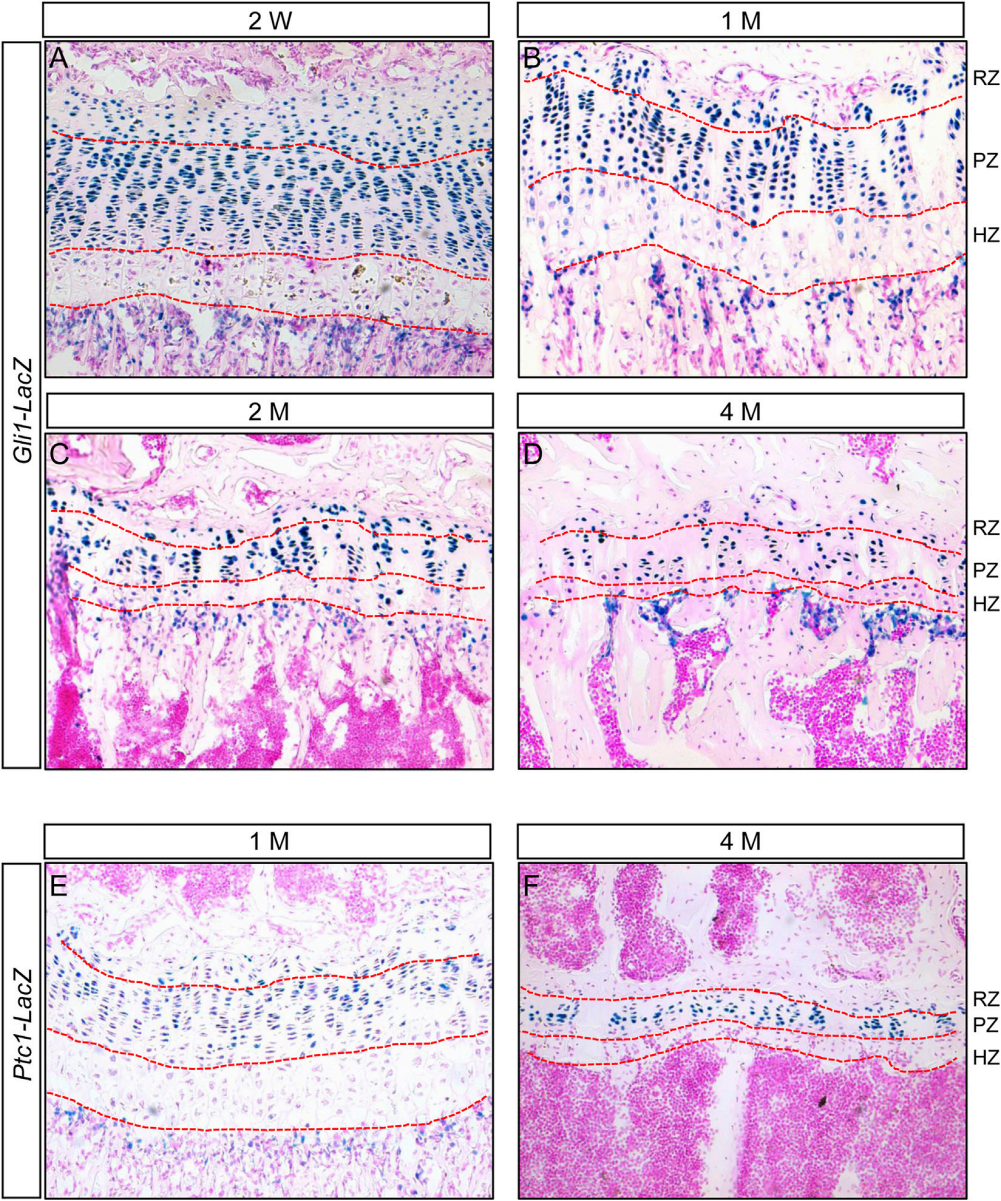
Hh signaling is active in columnar and resting chondrocytes, but downregulated in hypertrophic chondrocytes, in the epiphyseal growth plates of juvenile and adult mice **(A–D)** LacZ staining of longitudinal tibial sections from 2-week-old **(A)**, 1-month-old **(B)**, 2-month-old **(C)**, and 4-month-old **(D)** *Gli1-LacZ* reporter mice **(E,F)** LacZ staining of longitudinal tibial sections from 1-month-old **(E)** and 4-month-old **(F)** *Ptc1-LacZ* reporter mice. RZ, resting zone; PZ, proliferative zone, HZ, hypertrophic zone.

We previously showed that Hh signaling was activated in the chondrocytes of epiphyseal growth plates from 2- and 12-month-old mice (Zhang et al., 2022). To further characterize Hh-responsive cells in the epiphyseal growth plates during postnatal skeletal growth, we first examined the expression of *Gli1*, a transcriptional target of Hh signaling activity in juvenile and adult mice. We utilized *Gli1-LacZ* knock-in reporter mice, in which the bacterial LacZ gene is controlled by the endogenous Gli1 locus, and therefore its activity can be used as a readout of *Gli1* expression. LacZ staining of tibial sections from two-week-old *Gli1-LacZ* reporter mouse revealed strong *Gli1-LacZ* activity in nearly all chondrocytes in the columnar proliferative zone (PZ) (Figure 1A). Similarly, the vast majority of chondrocytes within the resting zone (RZ), a region that contains growth plate skeletal stem cells, were also LacZ-positive, although the LacZ signal in these cells tended to be slightly weaker than columnar chondrocytes at this stage ((Figure 1A). In contrast, the hypertrophic zone (HZ) exhibited significantly less LacZ-positive cells as well as weaker LacZ signal in these positive cells (Figure 1A). The similar LacZ expression patterns were also observed in the tibial growth plates of 1-, 2-, and 4-month-old *Gli1-LacZ* mice (Figures 1B–D). As the epiphyseal growth plates matured from 2 weeks to 4 months of age, the numbers of LacZ-positive chondrocytes in both resting and columnar zones tended to decrease gradually (Figures 1A–D). To confirm the distribution of Hh-responsive cells in the epiphyseal growth plates, we then monitored the expression of *Ptc1*, another target gene of Hh signaling, using *Ptc1-LacZ* knock-in reporter mice. Similar to the results from *Gli1-LacZ*, strong LacZ activity was detected in most of columnar chondrocytes and some resting chondrocytes, whereas almost no LacZ expression was observed in hypertrophic zones, in the tibial growth plates from 1-and 4-month-old *Ptc1-LacZ* mice (Figures 1E,F). Collectively, these results demonstrated that the columnar and resting chondrocytes are major Hh-responsive cells in the epiphyseal growth plates of juvenile and adult mice.

### Chondrocyte-specific deletion of *Smo* in juvenile mice led to defects in epiphyseal growth plates and limb elongation

**FIGURE 2:**
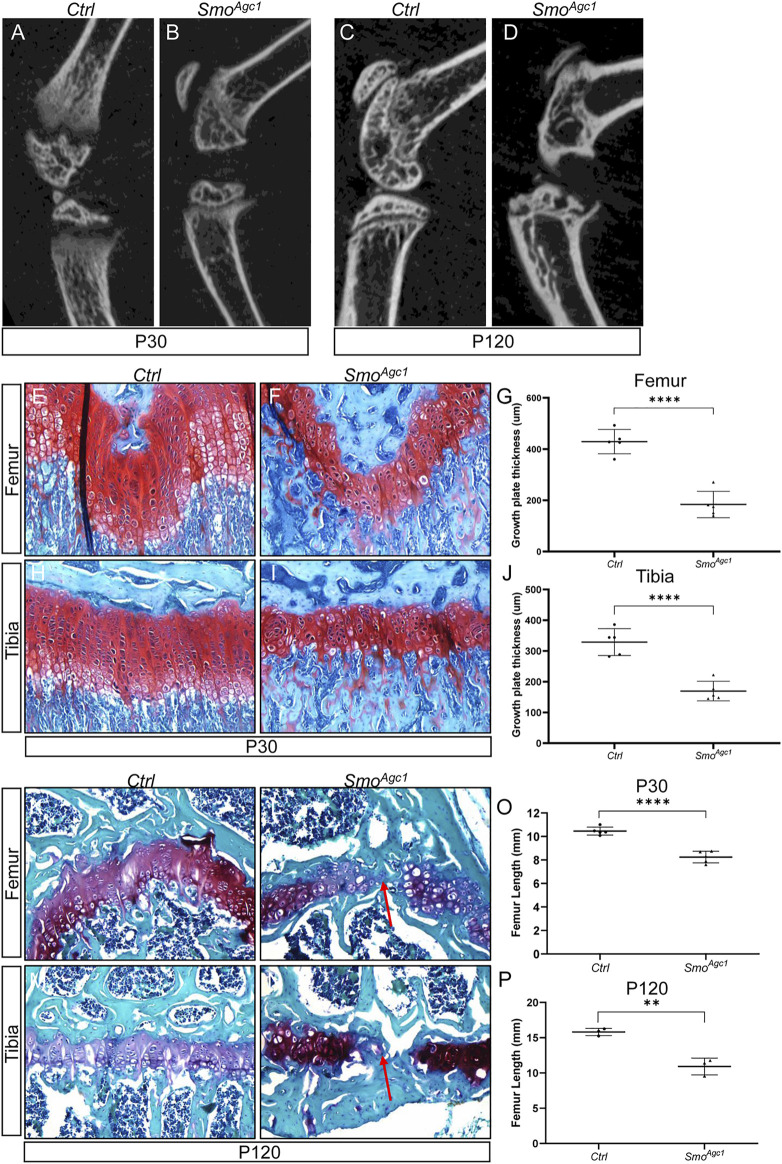
Chondrocyte-specific deletion of *Smo* in juvenile mice led to defects in epiphyseal growth plate and limb elongation **(A–D)** Representative uCT slices of the knee joint region in *Agc1-CreER* ^*T2*^ *; Smo* ^*f/f*^ (*Smo* ^*Agc1*^) **(A,C)** and control mice (Ctrl) **(B,D)** treated with tamoxifen at P14-P18 and harvested at P30 **(A,B)** or P120 **(C,D) (E,F)** Safranin O staining of distal femoral growth plates from P30 *Ctrl* and *Smo* ^*Agc1*^ mice **(G)** Quantification of average thickness of distal femoral growth plates **(H,I)** Safranin O staining of proximal tibial growth plates from P30 *Ctrl* and *Smo* ^*Agc1*^ mice **(J)**. Quantification of average thickness of proximal tibial growth plates (**K–N)** Safranin O staining of growth plates in distal femurs **(K,L)** or proximal tibiae **(M,N)** from P120 *Ctrl* and *Smo* ^*Agc1*^ mice. Red arrows indicated the fusion of the growth plates in the mutant distal femurs and proximal tibiae **(O,P)**. Measurement of average lengths of femurs from *Ctrl* and *Smo* ^*Agc1*^ mice at P30 **(O)** and P120 **(P)**. All quantitative data were shown as individual data points from each mouse together with mean ± standard deviation. *p* values were obtained by Student’s t-test and degrees of statistical significance were designated by different numbers of asterisk (*) in the figures: *, *p < 0.05*; **, *p < 0.01*; ***, *p < 0.001*; ****, *p < 0.0001*.

Since Hh signaling is activated in growth plate chondrocytes of juvenile and adult mice, we next investigated whether Hh-responsiveness in these cells was required for maintenance of epiphyseal growth plates after onset of SOC formation. Smoothened *(Smo)* is a G protein-coupled receptor essential for cells to respond to Hh signals. We therefore blocked Hh responsiveness in chondrocytes of juvenile mice by genetically removing *Smo* gene with tamoxifen-inducible *Agc1-CreER* ^*T2*^ allele. By using the *R26-tdTomato* reporter mice, we first confirmed that *Agc1-CreER* ^*T2*^ can efficiently target growth plate chondrocytes (Supplementary Figure S1), when induced in mice at 2 weeks of ages, a stage when the SOC has largely formed. We then used tamoxifen to induce Cre activity in 2-week-old *Agc1-CreER* ^*T2*^; *Smo* ^*fl/fl*^ (hereafter *Smo* ^*Agc1*^) mice and examined the effects of *Smo* deletion on epiphyseal growth plates. μCT analyses of tamoxifen-induced *Smo* ^*Agc1*^ mice identified obvious defects in the growth plates of the proximal tibiae and distal femurs. At P30 (12 days after tamoxifen injections), the growth plates were clearly visible in radiographical images of tibiae and femurs from both *Smo* ^*Agc1*^ and control mice (Figures 2A,B). However, their sizes appeared to be significantly reduced in mutant mice, when compared to control mice (Figures 2A,B). By P120, the growth plates from mutant mice exhibited a variable degree of defects. In the most severely-affected mutants, the growth plates of tibiae and femurs were both prematurely fused (Figures 2C,D). By contrast, the growth plates from control mice were still recognizable at this stage (Figure 2C), although became narrower compared to those at P30. To confirm these findings, we next performed histological analyses of long bones from *Smo* ^*Agc1*^ and control mice at P30 and P120 (12 and 102 days from the last dose of tamoxifen, respectively). Safranin O staining revealed that *Smo* deletion progressively impaired morphology and organization of growth plate chondrocytes in both tibiae and femurs (Figures 2E–J). Proliferating chondrocytes in control mice were arranged in longitudinal columns at P30, whereas this columnar organization of chondrocytes was notably disrupted at P30. Moreover, the composition of hypertrophic layer of chondrocytes were significantly reduced at this stage (Supplementary Figure S2). By P120, the integrity of growth plates in mutant mice was severely impaired (Figures 2K–N). In particular, the growth plates in distal femurs from mutant mice were all prematurely fused. Some growth plates from proximal tibiae were also similarly affected. Collectively, these results demonstrated that Hh signaling is cell-autonomously required for maintenance of the epiphyseal growth plates in juvenile mice.

To determine whether the changes in growth plate caused by *Smo* deletion had detrimental consequences on overall limb growth and elongation, we performed μCT-based analyses of femur length of *Smo* ^*Agc1*^ and control mice at different ages. At P30, femurs of mutant mice were shortened by 21%, when compared to those of control littermates (Figure 2O). By 120, this reduction in femur length was further enlarged to 30% (Figure 2P). Together, our results indicated that disruption of Hh signaling in growth plate chondrocytes of juvenile mice not only caused growth plate defects, but also impaired limb elongation.

### Chondrocyte-specific deletion of *Smo* in juvenile mice led to reduced chondrocyte proliferation and increased chondrocyte apoptosis

**FIGURE 3:**
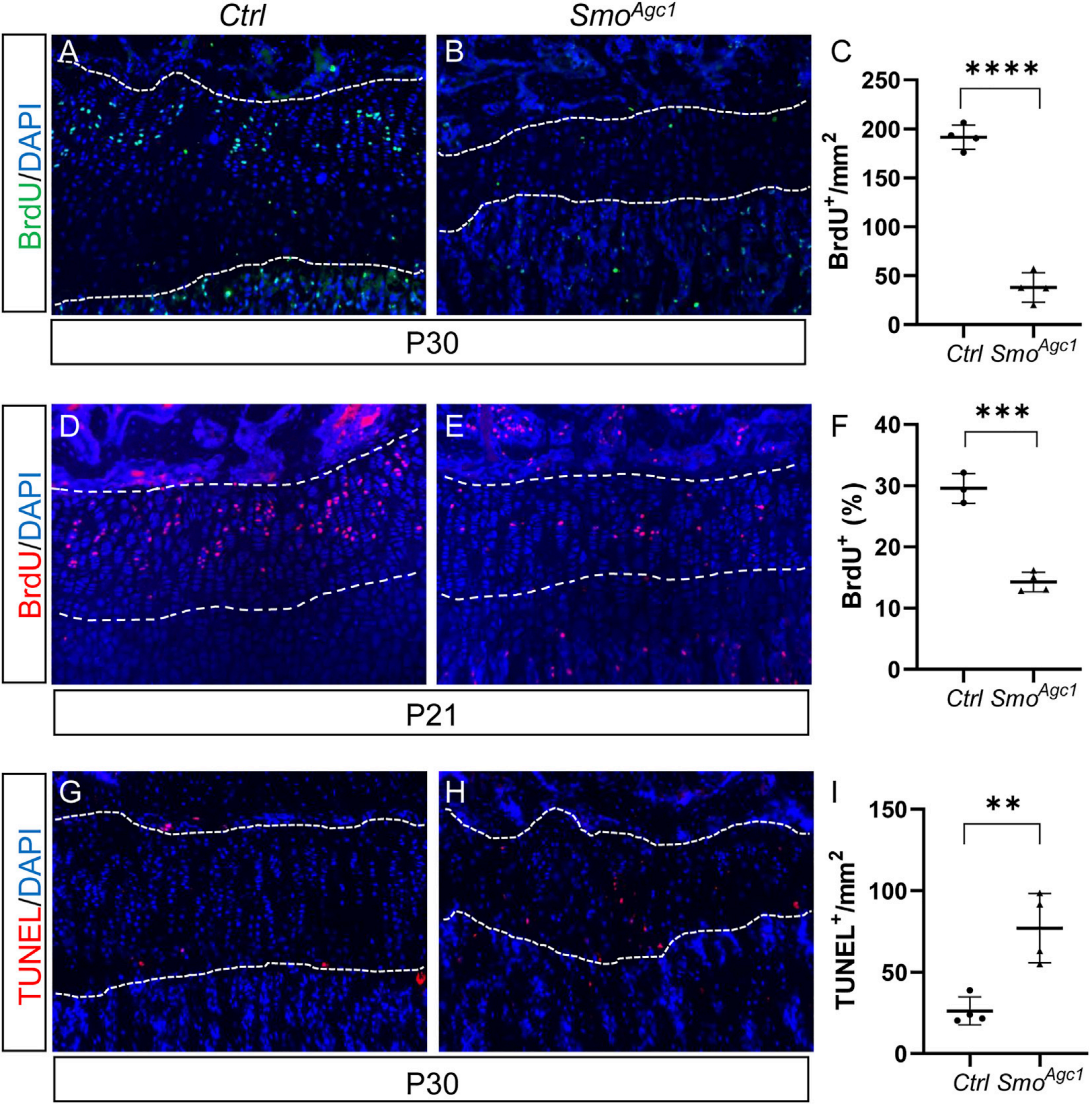
Chondrocyte-specific deletion of *Smo* in juvenile mice led to reduced chondrocyte proliferation and increased chondrocyte apoptosis **(A,B)** BrdU staining of proximal tibial growth plates from P30 *Ctrl* **(A)** and *Smo* ^*Agc1*^ mice **(B)**. BrdU-labeled cells were shown in green and nuclei were stained in blue **(C)** Quantification of BrdU-positive cells normalized to areas of proximal tibial growth plates in P30 *Ctrl* and *Smo* ^*Agc1*^ mice **(D,E)** BrdU staining of proximal tibial growth plates from P21 *Ctrl* **(D)** and *Smo* ^*Agc1*^ mice **(E)**. BrdU-labeled cells were shown in red and nuclei were stained in blue **(F)** Quantification of the percentage of BrdU-positive cells in columnar zones of proximal tibial growth plates from P21 *Ctrl* and *Smo* ^*Agc1*^ mice **(G,H)** TUNEL assay of proximal tibial growth plates from P30 *Ctrl* **(G)** and *Smo* ^*Agc1*^ mice **(H)**. Apoptotic cells were shown in red and nuclei were counterstained in blue **(I)** Quantification of TUNEL-positive cells normalized to areas of proximal tibial growth plates in P30 *Ctrl* and *Smo* ^*Agc1*^ mice. All mice were treated with 5 daily doses of tamoxifen starting at P14. All quantitative data were shown as individual data points from each mouse together with mean ± standard deviation. *p* values were obtained by Student’s t-test and degrees of statistical significance were designated by different numbers of asterisk (*) in the figures: *, *p < 0.05*; **, *p < 0.01*; ***, *p < 0.001*; ****, *p < 0.0001*.

Chondrocytes proliferation and survival are important contributors of growth plate maintenance in juvenile mice. To assess how deletion of the *Smo* in juvenile chondrocytes caused the growth plate defects, we first examined status of chondrocyte proliferation in *Smo* mutant mice at 12 days after tamoxifen administration. BrdU staining of tibial sections revealed abundant BrdU-positive chondrocytes in the growth plates of control mice (Figure 3A). By contrast, only a few proliferating cells were detected in the mutant growth plates at this stage (Figure 3B). Histomorphometric analysis confirmed that the number of proliferative chondrocytes, when normalized to areas of growth plates, was dramatically decreased in mutant growth plate when compared with controls (Figure 3C). Since a morphological recognizable columnar zone was largely absent in the growth plates of P30 mutants (Figure 2), it was infeasible to calculate the percentage of BrdU-positive columnar chondrocytes at this stage. Therefore, we examined chondrocyte proliferation at an earlier time when the mutant growth plates still contained the morphologically distinct columnar zones. The results showed that the percentage of BrdU-positive chondrocytes in the columnar region were significantly reduced in P21 *Smo* ^*Agc1*^ mice when compared to their littermate controls (Figures 3D–F). Collectively, these results demonstrated that Hh signaling is cell-autonomously required for chondrocyte proliferation.

Next, we evaluated whether increased chondrocyte apoptosis contributed to growth plate abnormality. To this end, we performed TUNEL assays on tibial sections from *Smo* ^*Agc1*^ and control mice at 12 d after tamoxifen administration. In line with the fact that apoptosis normally occurs in terminally differentiated hypertrophic chondrocytes, TUNEL-positive cells were detected at the chondro-osseous junction but were largely absent in other regions of the growth plate in control mice (Figure 3G). By contrast, apoptotic cells were observed not only at the chondro-osseous junction, but also in the middle of growth plates in mutants (Figure 3H). Quantitative analysis confirmed an increase in number of TUNEL-positive cells in the growth plates of mutant mice, when compared to control mice (Figure 3I). Thus, the growth chondrocytes in juvenile mice, unlike their counterparts at embryonic stages, requires direct Hh input to maintain their survival.

### Chondrocyte-specific deletion of *Smo* in juvenile mice led to premature hypertrophic differentiation

**FIGURE 4:**
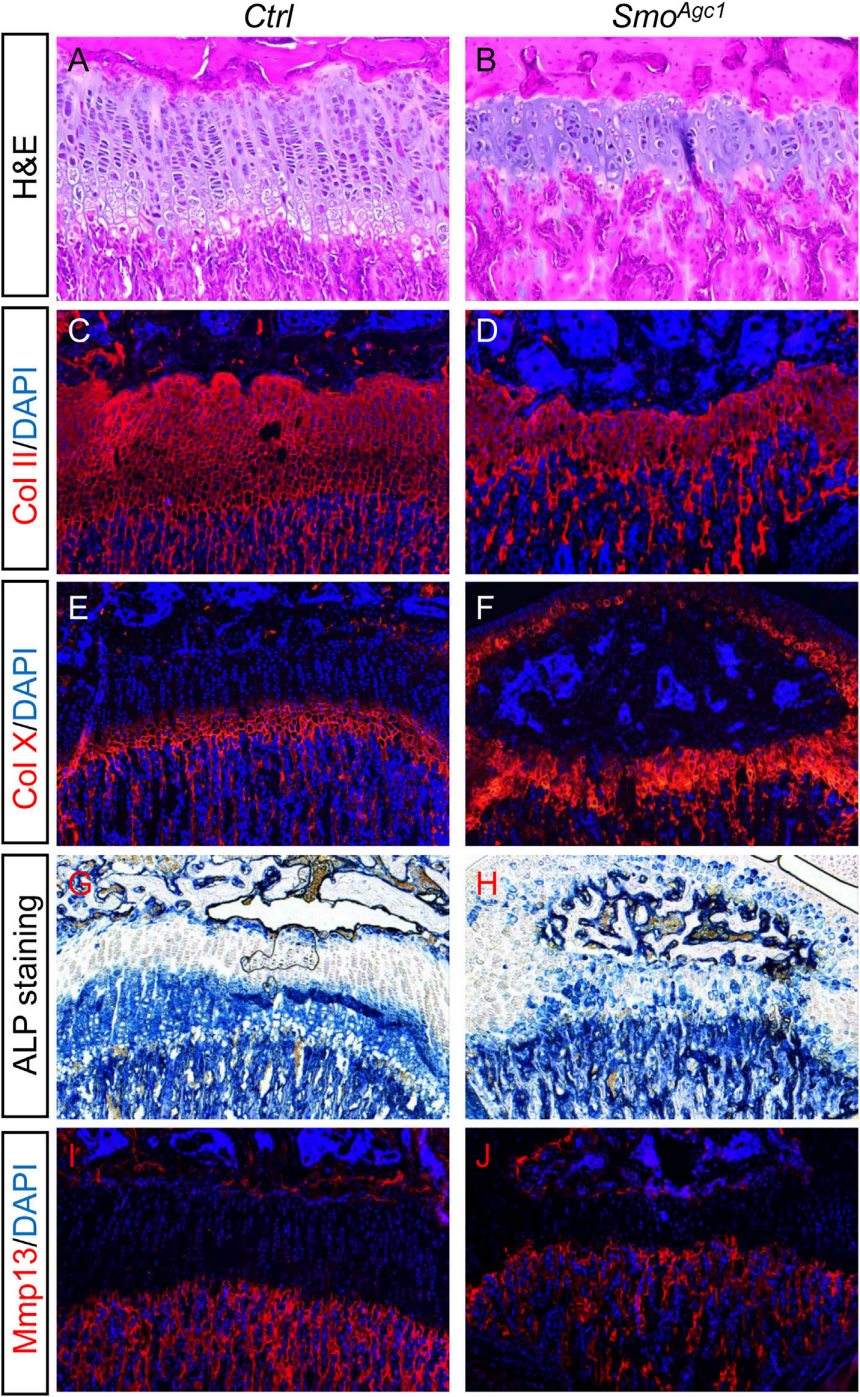
Chondrocyte-specific deletion of *Smo* in juvenile mice led to premature hypertrophic differentiation **(A,B)** H&E staining of the proximal tibial growth plates from P30 *Ctrl* **(A)** and *Smo* ^*Agc1*^ mice **(B) (C–F)** Immunofluorescent staining of collagen type Ⅱ (Col II) **(C,D)** and collagen type X (Col X) **(E,F)** on frozen sections of the proximal tibial growth plates from P30 *Ctrl* **(C,E)** and *Smo* ^*Agc1*^ **(D,F)** mice **(G,H)** ALP staining of the proximal tibial growth plates from P30 *Ctrl* **(G)** and *Smo* ^*Agc1*^ mice **(H) (I,J)** Immunofluorescent staining of Mmp13 on frozen sections of the proximal tibial growth plates from P30 *Ctrl* **(I)** and *Smo* ^*Agc1*^ **(J)** mice. All mice were treated with 5 daily doses of tamoxifen starting at P14.

Postnatal growth plate is maintained not only by chondrocyte proliferation and survival, but also by chondrocyte differentiation. We therefore investigated whether chondrocyte differentiation was altered by *Smo* deletion. Histological analysis of the proximal tibial growth plates of *Smo* ^*Agc1*^ mice revealed that almost no chondrocytes with enlarged morphology were detected at P30 (Figures 4A,B). Immunofluorescent staining showed that nearly all chondrocytes in the mutant growth plates expressed collagen type Ⅱ (Col II) and collagen type X (Col X) proteins (Figures 4C–F), the latter of which is normally restricted to the lower hypertrophic zone of the growth plates. Moreover, ALP, a marker normally expressed by all stages of hypertrophic chondrocytes, was ectopically expressed in chondrocytes throughout the growth plates in mutant mice (Figures 4G,H). In contrast, expression of Mmp13, a marker for terminal differentiated hypertrophic chondrocytes, was similar between controls and mutants (Figures 4I,J). Together, these results demonstrated that postnatal deletion of *Smo* from chondrocytes accelerated earlier steps of hypertrophic differentiation, but did not affect terminal stages of chondrocyte hypertrophy.

### Chondrocyte-specific deletion of *Sufu* in juvenile mice caused a transient expansion of growth plates followed by their premature fusion

**FIGURE 5:**
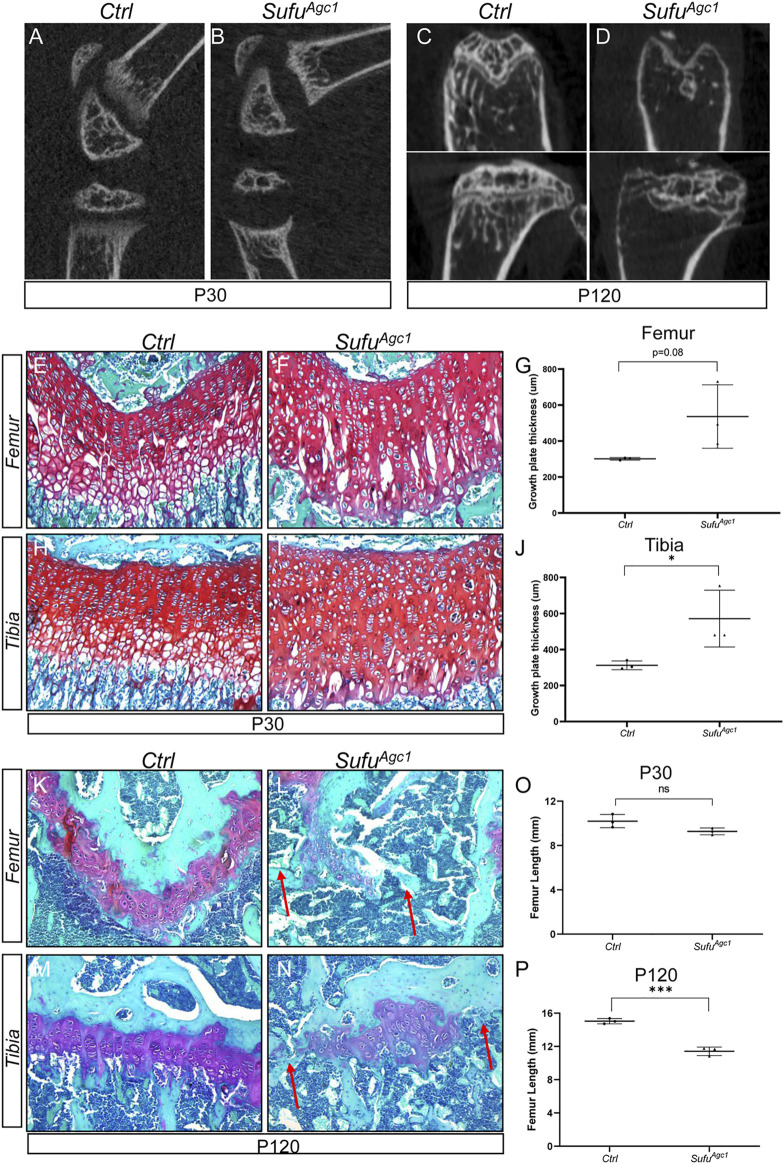
Chondrocyte-specific deletion of *Sufu* in juvenile mice caused a transient expansion of growth plates followed by their premature fusion **(A–D)** Representative uCT slices of growth plates in *Agc1-CreER* ^*T2*^ *; Sufu* ^*f/f*^ (*Sufu* ^*Agc1*^) **(A,C)** and control mice (*Ctrl*) **(B,D)** at P30 **(A,B)** or P120 **(C,D) (E,F)** Safranin O staining of distal femoral growth plates from P30 *Ctrl* and *Sufu* ^*Agc1*^ mice **(G)** Quantification of average thickness of distal femoral growth plates from P30 *Ctrl* and *Sufu* ^*Agc1*^ mice **(H,I)** Safranin O staining of proximal tibial growth plates from P30 *Ctrl* and *Sufu* ^*Agc1*^ mice **(J)** Quantification of average thickness of proximal tibial growth plates from P30 *Ctrl* and *Sufu* ^*Agc1*^ mice **(K–N)** Safranin O staining of growth plates in distal femurs **(K,L)** or proximal tibiae **(M,N)** from P120 *Ctrl* and *Sufu* ^*Agc1*^ mice. Red arrows indicated the fusion of the growth plates in the mutant distal femurs and proximal tibiae **(O,P)**. Measurement of average lengths of femurs from *Ctrl* and *Sufu* ^*Agc1*^ mice at P30 **(O)** and P120 **(P)**. All quantitative data were shown as individual data points from each mouse together with mean ± standard deviation. *p* values were obtained by Student’s t-test and degrees of statistical significance were designated by different numbers of asterisk (*) in the figures: *, *p < 0.05*; **, *p < 0.01*; ***, *p < 0.001*; ****, *p < 0.0001*.

Hh signaling is normally tightly regulated, whereas either inactivation or hyperactivation could lead to defects in tissue development and homeostasis. Therefore, we next tested whether restraint of Hh activity level was critical for epiphyseal growth plate maintenance and limb elongation in juvenile mice. To this end, we conditionally deleted *Sufu*, a negative regulator of Hh signaling, to forcedly activate Hh signaling in growth plate chondrocytes of juvenile mice. We administered 5 daily doses of tamoxifen to *Agc1-CreER* ^*T2*^; *Sufu* ^*fl/fl*^ (hereafter *Sufu* ^*Agc1*^) mice starting at P14 and performed radiographic and histological analyses of epiphyseal growth plates at P30 and P120. μCT imaging of tibiae and femurs revealed that the growth plates were abnormally formed in *Sufu* ^*Agc1*^ mice (Figures 5A–D). At P30, the growth plates of tibia and femurs in mutant mice, as shown by the translucent area between primary and secondary ossification centers in the μCT images, were obviously expanded compared with control mice (Figures 5A,B). However, by P120, the expansion of growth plate was no longer observed in radiographical images of mutant mice that instead exhibited the complete or partial closure of growth plates (Figures 5C,D). To confirm these observations, we next performed histological analyses. Safranin O staining showed that *Sufu*-deficient mice had enlarged growth plates of both tibiae and femurs, compared to wild-type littermates at P30 (Figures 5E–J). In addition, the morphology and organization of the growth plates in mutant mice were strikingly different from controls. The control growth plates were composed of three morphologically distinct zones of chondrocytes: the round chondrocytes at the top resting zone, the flat column-forming chondrocytes in the middle zone, and the hypertrophic chondrocytes at the bottom zone (Figures 5E,H). By contrast, this orderly structure was lost in the mutant growth plate, which was instead occupied by a poorly-organized mass of chondrocytes with heterogenous morphology (Figures 5F,I). These changes appeared to negatively affect growth plates in the long term. At P120, the growth plates from mutant mice were prematurely closed, as evidenced by a significant loss of Safranin O-stained cartilages in the mutant growth plates (Figures 5K–N; Supplementary Figure S3). As a control, morphologically intact growth plates remained evident in both tibiae and femurs from tamoxifen-injected control mice (Figures 5K–N; Supplementary Figure S3). Thus, chondrocyte-specific deletion of *Sufu* in juvenile mice caused a transient expansion of growth plate followed by its premature fusion.

To evaluate the consequences of growth plates defects in *Sufu*-deficient mice on their overall limb growth and elongation, we also utilized μCT images to measure length of femurs from *Sufu* ^*Agc1*^ and control mice at P30 and P120. The results showed that femur length of P30 *Sufu* ^*Agc1*^ mice was slightly reduced when compared with those of control littermates, although this difference did not reach the statistical significance (Figure 5O). Moreover, femurs from mutant mice became significantly shorter than those of control mice by P120 (Figure 5P), indicating that loss of *Sufu* impaired limb elongation. Together, these results indicated that *Sufu*-mediated restraint of Hh activity level is critical for epiphyseal growth plate maintenance and limb elongation in juvenile mice.

### Chondrocyte-specific deletion of *Sufu* in juvenile mice impaired chondrocyte proliferation and survival

**FIGURE 6:**
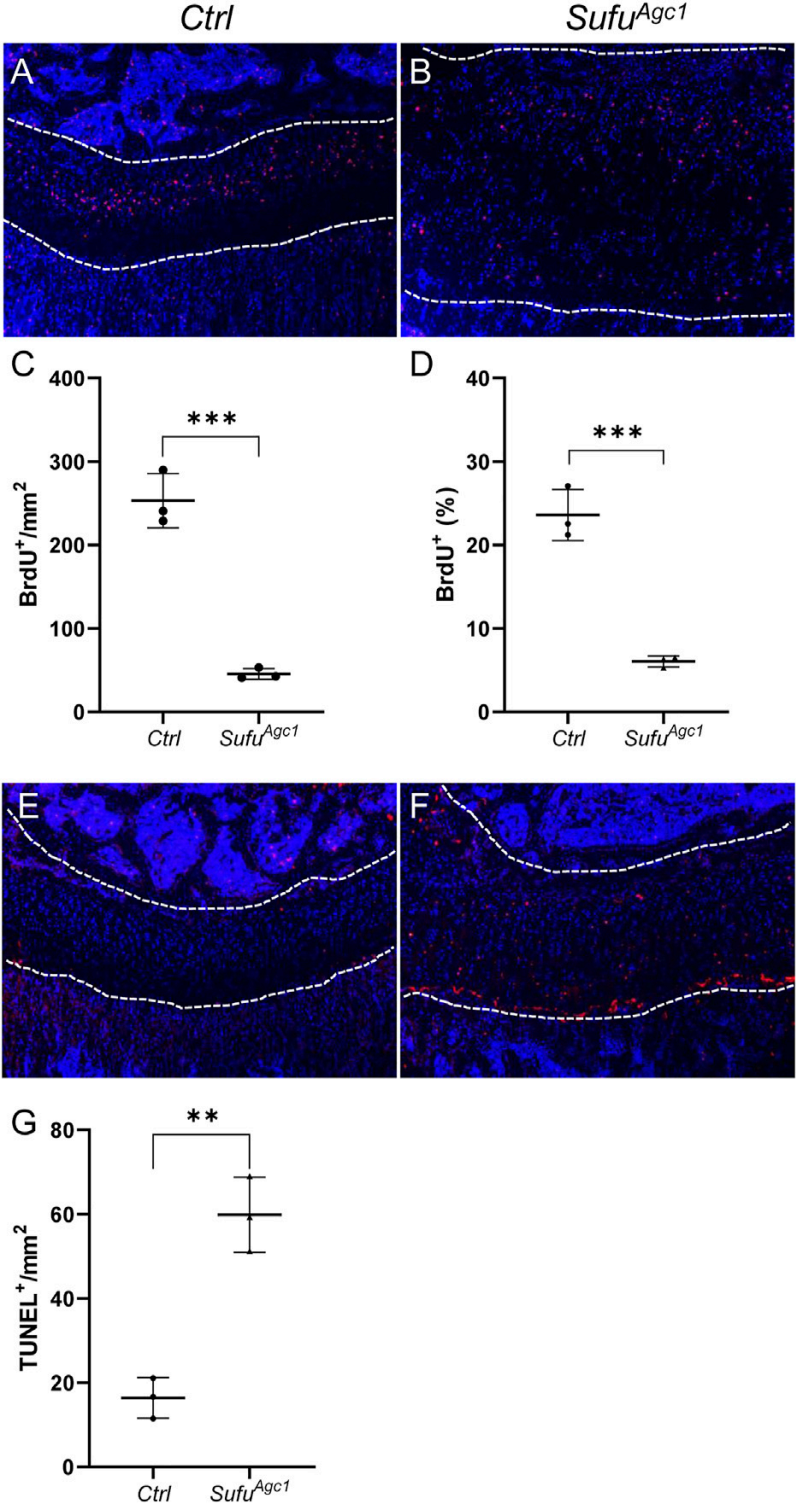
Chondrocyte-specific deletion of *Sufu* in juvenile mice impaired chondrocyte proliferation and promoted cell apoptosis **(A,B)** BrdU staining of proximal tibial growth plates from P30 *Ctrl* **(A)** and *Sufu* ^*Agc1*^mice **(B)**. BrdU-labeled cells were shown in red and nuclei were stained in blue **(C,D)** Quantification of the number **(C)** and percentage **(D)** of BrdU-positive cells in resting and columnar zones of proximal tibial growth plates from P30 *Ctrl* and *Sufu* ^*Agc1*^ mice **(E,F)** TUNEL assays of proximal tibial growth plates from P30 *Ctrl* **(E)** and *Sufu* ^*Agc1*^ mice **(F)**. Apoptotic cells were shown in red and nuclei were counterstained in blue **(G)** Quantification of TUNEL-positive cells normalized to areas of proximal tibial growth plates in P30 *Ctrl* and *Sufu* ^*Agc1*^ mice. All mice were treated with 5 daily doses of tamoxifen starting at P14. All quantitative data were shown as individual data points from each mouse together with mean ± standard deviation. *p* values were obtained by Student’s t-test and degrees of statistical significance were designated by different numbers of asterisk (*) in the figures: *, *p < 0.05*; **, *p < 0.01*; ***, *p < 0.001*; ****, *p < 0.0001*.

To assess the effect of *Sufu* deletion on chondrocyte proliferation, we performed BrdU assays at 12 d after tamoxifen injection. As expected, BrdU-positive proliferating chondrocytes were mainly localized in the columnar zone of the control growth plates (Figure 6A). By contrast, these cells were scattered across the entire growth plates from mutant mice (Figure 6B). Surprisingly, despite the remarkably increased numbers of total chondrocytes, the number of proliferative chondrocytes were significantly decreased in mutant growth plate compared to the controls (Figure 6C). Consequently, the rate of chondrocyte differentiation, as calculated by the percentage of proliferative chondrocytes out of total immature chondrocytes, was significantly reduced in *Sufu*-deficient mice (Figure 6D), indicating that *Sufu*-mediated restraint of Hh signaling in chondrocytes is critical for their proliferation. Next, we performed TUNEL assays on tibial sections from P30 *Smo* ^*Agc1*^ and control mice to evaluate the impact of *Sufu* deletion on chondrocyte apoptosis. As expected, apoptotic cells were mainly localized at the border between the growth plate and primary spongiosa in control mice (Figure 6E). In contrast, TUNEL-positive cells were distributed throughout the enlarged growth plates in mutants (Figure 6F). Quantitative analysis further revealed that the number of TUNEL-positive chondrocytes was strikingly induced by *Sufu* deletion (Figure 6G), indicating that activity of Hh signaling in chondrocytes needs to be tightly restrained by *Sufu* for their survival. Collectively, these results demonstrated that *Sufu*-mediated restraint of Hh signaling in chondrocytes plays an essential role in promoting chondrocyte proliferation and suppressing chondrocyte apoptosis in juvenile mice.

### Chondrocyte-specific deletion of *Sufu* in juvenile mice inhibited hypertrophic differentiation

**FIGURE 7:**
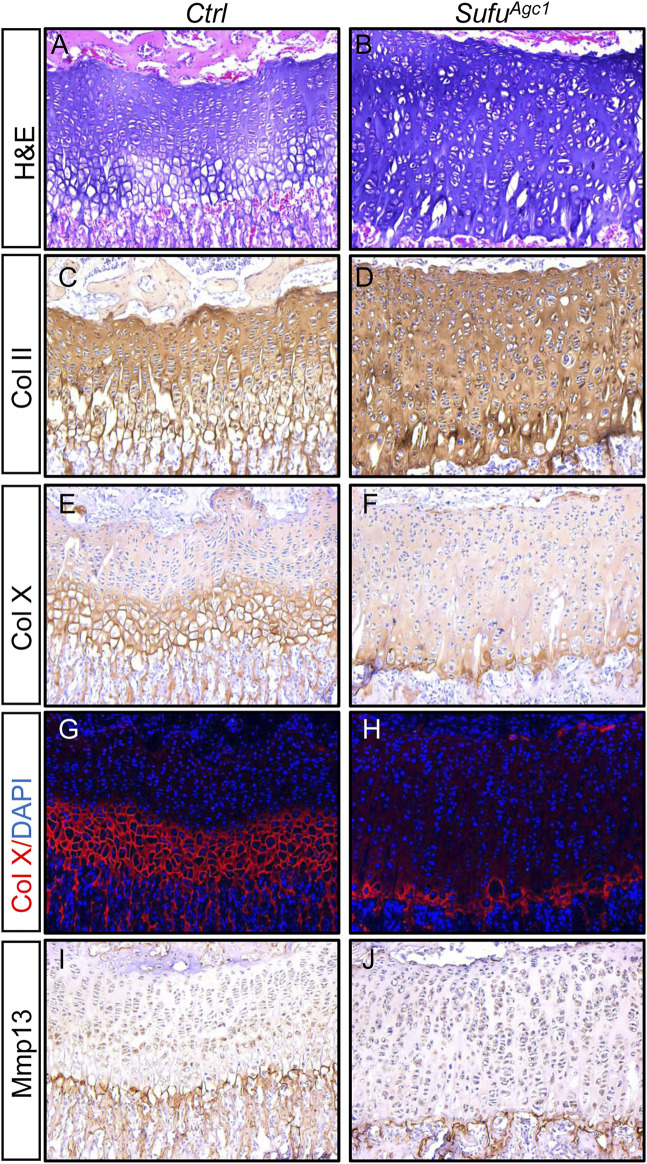
Chondrocyte-specific deletion of *Sufu* in juvenile mice inhibited hypertrophic differentiation **(A,B)** H&E staining of the proximal tibial growth plates from P30 *Ctrl* **(A)** and *Sufu* ^*Agc1*^ mice **(B) (C–F)** Immunohistochemical staining of collagen type Ⅱ (Col II) **(C,D)** and collagen type X (Col X) **(E,F)** on frozen sections of the proximal tibial growth plates from P30 *Ctrl* **(C,E)** and *Sufu* ^*Agc1*^ **(D,F)** mice **(G,H)** Immunofluorescent staining of Col X on frozen sections of the proximal tibial growth plates from P30 *Ctrl* **(G)** and *Sufu* ^*Agc1*^ **(H)** mice **(I,J)** Immunohistochemical staining of Mmp13 on frozen sections of the proximal tibial growth plates from P30 *Ctrl* **(I)** and *Sufu* ^*Agc1*^ **(J)** mice. All mice were treated with 5 daily doses of tamoxifen starting at P14 and analyzed at indicate time.

Next, we evaluated the status of chondrocyte differentiation in *Sufu*-deficient mice. H&E staining of tibial growth plates revealed that although the mutant growth plates have significantly more cells than controls, they did not appear to form morphologically distinct hypertrophic chondrocytes (Figures 7A,B), suggesting that chondrocyte-specific deletion of *Sufu* could suppress formation of hypertrophic chondrocytes. Furthermore, consistent with less BrdU-positive proliferating cells, no obvious increase in the number of flat column-forming cells was observed in the mutant growth plates (Figures 7A,B). Histomorphometrical analyses of tibial growth plates revealed that *Sufu* ablation led to increased composition of resting layers of chondrocytes, but reduced composition of hypertrophic zones without significantly altering the portion of columnar chondrocytes (Supplementary Figure S4). To confirm these observations at the molecular levels, we performed immunostaining of longitudinal sections from tibias harvested at P30 to examine the expression of chondrocyte differentiation markers. Growth plates from control mice exhibited high level of ColII protein in proliferative zones, but significantly lower level in hypertrophic zones (Figure 7C). In comparison, the mutant growth plates exhibited strong and uniform expression of Col II in the entire growth plate (Figure 7D). On the other hand, both IHC and IF staining showed that the number of cells expressing ColX was remarkably reduced in mutant growth plates when compared to controls (Figures 7E–H). Collectively, these morphological and molecular analyses demonstrated that postnatal deletion of *Sufu* from chondrocytes inhibited chondrocyte hypertrophy. However, unlikely ColⅡ and ColX, Mmp13 was similarly detected in the last row of the hypertrophic zone of growth plates from both controls and mutants (Figures 7I,J), indicating that terminal differentiation of chondrocytes was not altered by *Sufu* deletion. Thus, the expansion of the growth plate observed in *Sufu*-deficient mice is due to accumulation of immature chondrocytes partly attributable to impaired chondrocyte hypertrophy.

### *Osx-Cre*-mediated ablation of either *Smo* or *Sufu* in hypertrophic chondrocytes did not overtly affect growth plates

**FIGURE 8:**
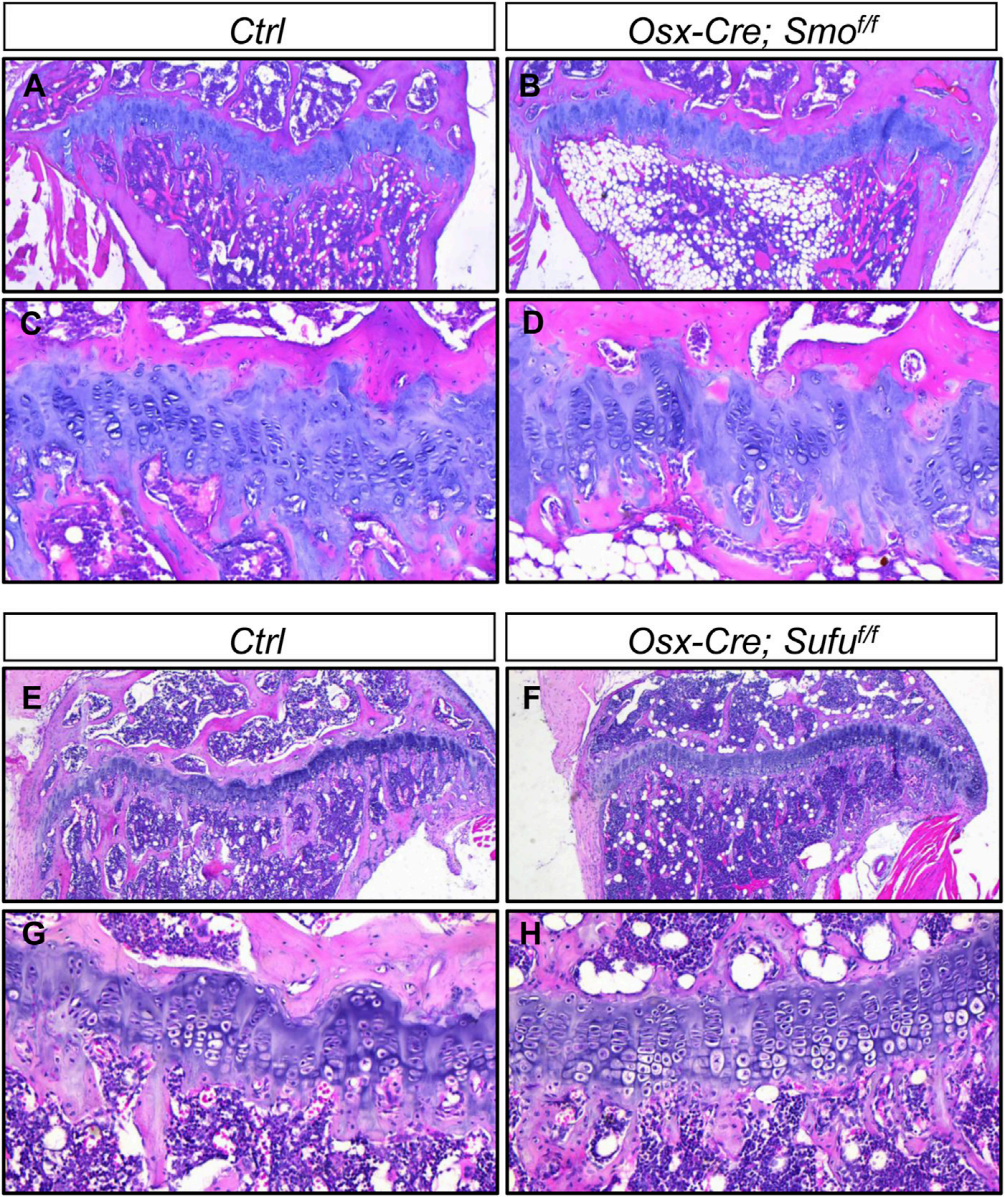
*Osx-Cre*-mediated ablation of either *Smo* or *Sufu* in hypertrophic chondrocytes did not overtly affect growth plates **(A–D)** H&E staining of the proximal tibial growth plates from P120 *Ctrl* **(A,C)** and *Osx-Cre; Smo* ^*f/f*^ mice **(B,D)** shown at low magnification **(A,B)** and higher magnification **(C,D) (E–H)** H&E staining of the proximal tibial growth plates from P90 *Ctrl* **(E,G)** and *Osx-Cre;Sufu* ^*f/f*^ mice **(F,H)** shown at low magnification **(E,F)** and higher magnification **(G,H)**.

Thus far, we have demonstrated that undisturbed Hh signaling in chondrocytes is essential for growth plate maintenance in juvenile mice. In contrast, a recent study showed that modulating Hh signaling by conditionally ablating either *Smo* or *Ptch1* in *Col10a1*-expressing hypertrophic chondrocytes did not impair growth plate cartilage (Wang et al., 2022), suggesting that Hh signaling might mainly function in immature chondrocytes. To further explore this possibility in our hand, we utilized *Osx-Cre* to ablate *Smo* or *Sufu* in prehypertrophic/hypertrophic chondrocytes, since we have previously showed that *Osx-Cre* can efficiently mediate recombination in these cells in postnatal growing mice (Zhang et al., 2022). In line with the finding by Wang et al., histological analyses of tibiae from 4-month-old *Osx-Cre; Smo* ^*f/f*^ (*Smo* ^*Osx*^) and their littermate controls revealed that *Osx-Cre*-mediated ablation of *Smo* did not overtly affect tibial growth plates (Figures 8A–D). Similarly, the enlarged or prematurely fused growth plates was not observed in tibial growth plates from 3-month-old *Osx-Cre*; *Sufu* ^*f/f*^ (*Sufu* ^*Osx*^) mice (Figures 8E–H). Together, our results demonstrated that Hh signaling mainly functions in immature chondrocytes, not but in hypertrophic chondrocytes, to maintain growth plates at the juvenile stage.

## Discussion

Genetic studies have established the cell-autonomous and cell-non-cell-autonomous roles of Hh signaling in regulating epiphyseal cartilage development (Ohba, 2020). However, a chondrocyte-specific requirement of Hh signaling for growth plate maintenance during juvenile growth was yet to be established. In the present study, we utilized genetic approaches to specifically inactivate or activate Hh signaling in growth plate chondrocytes of juvenile mice, and made two major findings:1) Hh signaling functions in immature chondrocytes to promote chondrocyte proliferation and survival, while inhibiting chondrocyte hypertrophy during juvenile life in mice; 2) *Sufu*-mediated restraint of Hh activity is critical for maintaining juvenile growth plates. This study provided the first genetic evidence to establish the essential cell-autonomous role of fine-tuned Hh signaling in regulating multiple aspects of growth plate chondrocytes in juvenile mice.

Previous studies have shown that the tibial growth plates were essentially closed at 8 days after 2 doses of LDE225 (administered daily from P22 to P23) (Koyama et al., 2021). By contrast, we found that *Agc1-CreER* ^*T2*^-mediated ablation of *Smo* in growth plates of 2-week-old mice only led to partial fusion of tibial growth plates by P120. The cause why transient inhibition of Hh signaling caused more deleterious effect on tibial growth plates than permanent ablation of *Smo* in chondrocytes is still unclear. One possibility is that *Agc1-CreER* ^*T2*^, when induced in mice at 2 weeks of age, was not efficient to ablate *Smo* in growth plate chondrocytes. As a result, *Smo* inhibitors suppressed Hh signaling activity in chondrocytes to a greater degree than *Smo* ablation, therefore leading to more severer growth plate defects. Alternatively, this difference may reflect the important role of Hh signaling in regulating chondrocyte production from chondroprogenitors outside the growth plate. In line with this possibility, Axin2-positive perichondrial cells in the Ranvier’s groove (RG) were shown to contribute to the growth plates during juvenile growth (Usami et al., 2019). Importantly, Hh signaling, as indicated by LacZ signal in *Gli1-LacZ* reporter mice, was also activated in the RG (data not shown). Thus, it is possible that treatment with *Smo* inhibitors, but not chondrocyte-specific ablation of *Smo*, suppressed Hh signaling in the RG, and therefore undermined their ability to contribute to growth plates. Further studies are warranted to explore these possibilities.

Previous pharmacological studies reported inconsistent results about the role of Hh signaling in regulating chondrocyte hypertrophy during the juvenile period of growth. Premature chondrocyte hypertrophy was detected in mice treated with Smo antagonists HhAntag for 5 days (P10-14) or LDE225 (sonidegib) for 2 days (P22-P23), but not in mice treated with 6 doses of Smo antagonist vismodegib (Kimura et al., 2008; Newton et al., 2019; Koyama et al., 2021). In this study, we showed that chondrocyte-specific deletion of Smo in 2-week-old mice led to premature hypertrophic differentiation at P30. Therefore, our results strongly supported an essential role of Hh signaling in inhibiting chondrocyte hypertrophy during juvenile growth. This inhibitory function of Hh signaling in juvenile mice is consistent with its roles during cartilage development where Hh signaling restricts chondrocyte hypertrophy via an indirect mechanism involving *PTHrP* expression in periarticular cells (Long et al., 2001). Interestingly, *PTHrP* is also expressed in the resting chondrocytes in juvenile mice, and conditional ablation of *PTHrP* receptor in postnatal chondrocytes results in accelerated chondrocyte hypertrophy (Hirai et al., 2011). Thus, it is likely that Hh signaling suppresses hypertrophic differentiation of chondrocytes by promoting *PTHrP* expression in the resting chondrocytes. In addition to this inhibitory role, Ihh signaling can directly promote chondrocyte hypertrophy independently of PTHrP during embryonic skeletal development (Mak et al., 2008). It will be interesting to determine whether such a mechanism also functions in the postnatal cartilage.

Our LacZ staining results showed that hypertrophic chondrocytes of *Gli1*-and *Ptc1-LacZ* reporter mice had very low levels of *LacZ* expression, suggesting a low level of Hh signaling activity in these cells. However, despite ectopic expression of markers of hypertrophic chondrocytes in resting/proliferating chondrocytes, *Smo* ^*Agc1*^ mice developed a clear loss of hypertrophic zones. The similar results were also observed in mice treated with LDE225 (sonidegib) for 2 days (P22-P23) (Koyama et al., 2021). These seemingly conflicting results could be explained by the fact that Hh signaling promotes chondrocyte proliferation and survival, but restricts their maturation. Thus, while inactivation of Hh signaling accelerates hypertrophic differentiation of proliferating chondrocytes, it also causes a rapid depletion of growth plate chondrocytes due to reduced proliferation and increased apoptosis. As a result, the growth plates in *Smo* ^*Agc1*^ mice cannot continuously generate sufficient hypertrophic chondrocytes, and subsequently the hypertrophic zones in these mice are eroded rapidly by the chondroclasts.

Our data showed that ablation of either *Smo* or *Sufu* in chondrocytes led to impaired chondrocyte proliferation and survival, which could partially explain the premature fusion of growth plates in these mice. Similar phenomenon was also observed in pharmacological studies, in which both *Smo* agonist (SAG) and inhibitor (LDE225) treatments significantly decreased number of columns in the proliferating zone (Mizuhashi et al., 2018). Currently, the reason why inactivation and over-activation of Hh signaling exerted the same detrimental effects on chondrocytes is still unclear. Previous studies have shown that chondrocyte proliferation can be positively and negatively regulated by IGF1 and FGF18/FGFR3 signaling pathways, whereas BMP signaling is critical for both chondrocyte proliferation and survival (Hallett et al., 2019). Interestingly, Hh signaling has been shown to interact with the above signaling pathways in certain contexts (Hallett et al., 2019). However, it is unclear whether the similar interactions occur in the context of chondrocyte proliferation or survival in juvenile mice. Similarly, Hh signaling can directly promote chondrocyte proliferation during growth plate development by increasing expression of cyclin D1, a regulator of cell cycle progression (Hallett et al., 2019). However, whether this mechanism works in juvenile growth plates remains to be tested. Despite these uncertainties, our results clearly indicated that Hh signaling needs to be tightly regulated for its proper function in chondrocytes.

One limitation of our study is that we did not evaluate the effects of Hh manipulation on growth plate skeletal stem cells (gpSSC). Previous studies have suggested that Shh in the bony epiphyses may function as a niche to maintain SSC within the resting zone (Newton et al., 2019). However, the genetic evidence is still needed to support such a role of Hh signaling. Another limitation of our study is that we did not thoroughly investigate the downstream mechanism that mediates the effects of Hh signaling on chondrocytes. However, we did generate compound knockout mice with genetic deletion of both *Smo* and *Sufu* in chondrocytes, and found that these mice exhibited the same growth plate phenotypes as *Sufu* knockout mice (data not shown). Given that *Sufu* mainly function as a modulator of Gli activator and repressor, these genetic results suggested that Hh-*Smo* signaling likely regulates growth plate chondrocytes through Gli proteins. Clearly, the relative contribution of different Gli proteins remains to be determined. In summary, while further investigation is surely needed to address the above questions, the present study clearly demonstrated that the fine-tuned Hh signaling activity in immature chondrocytes is essential for epiphyseal growth plate maintenance and limb elongation in juvenile mice.

## Data Availability

The raw data supporting the conclusions of this article will be made available by the authors, without undue reservation.
