# Supplementary figures and images for: Suppressor of fused-restrained Hedgehog signaling in chondrocytes is critical for epiphyseal growth plate maintenance and limb elongation in juvenile mice
Source: Front Cell Dev Biol. 2022 Sep 2;10:997838. doi: 10.3389/fcell.2022.997838 (PMC9479194; doi:10.3389/fcell.2022.997838)

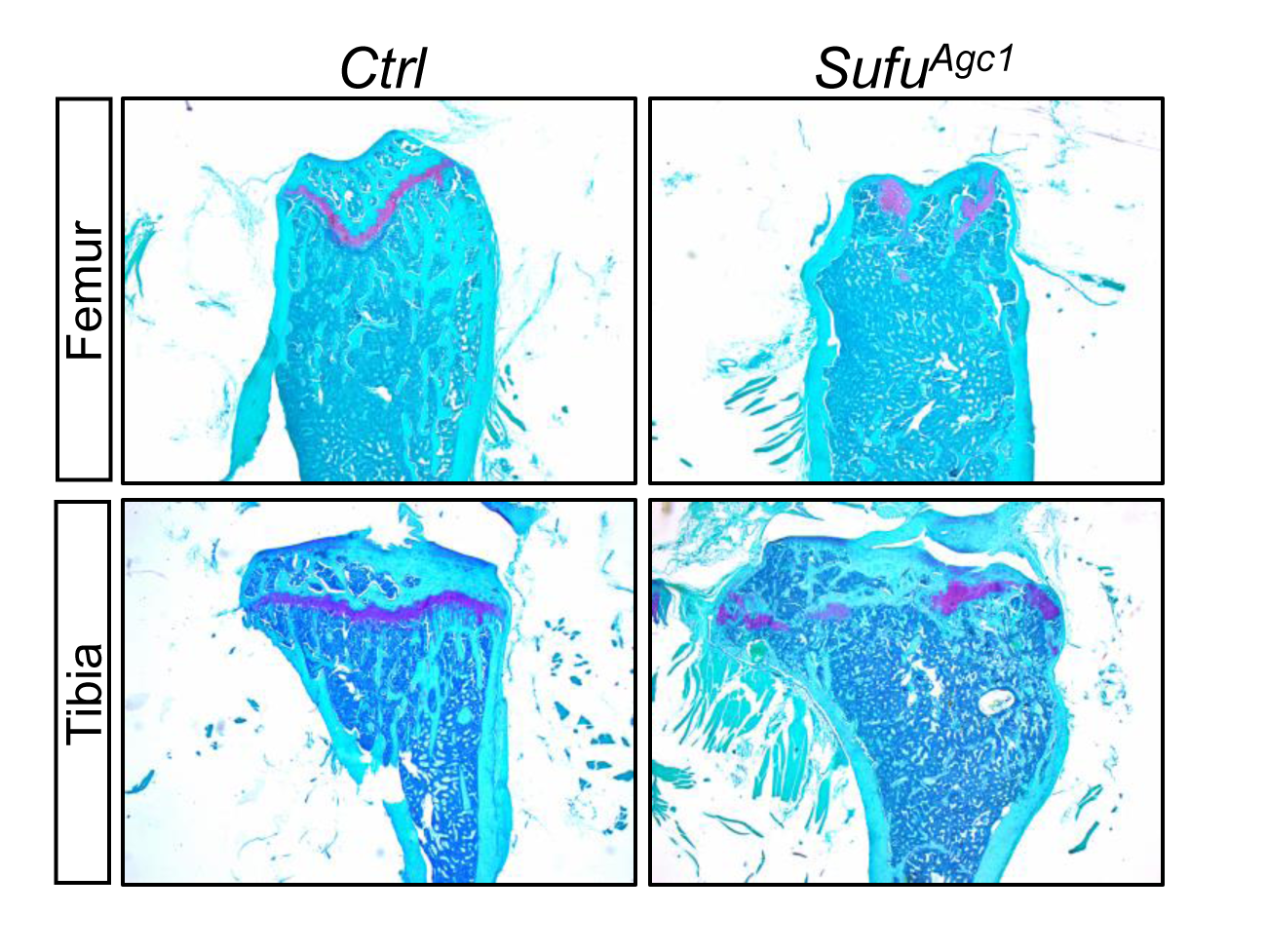

Supplement: Supplementary file 1 [file Image3.TIF]

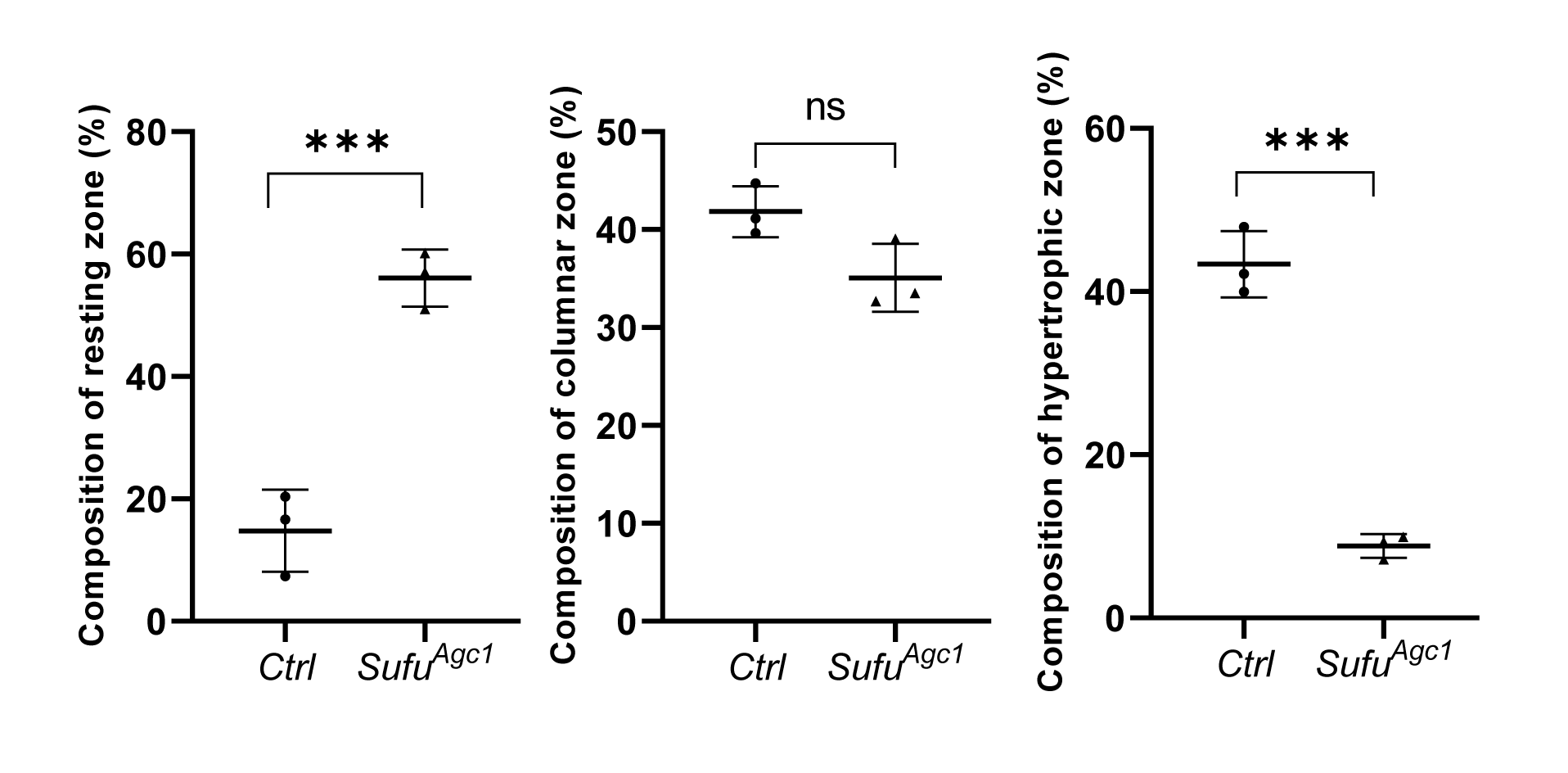

Supplement: Supplementary file 2 [file Image4.TIF]

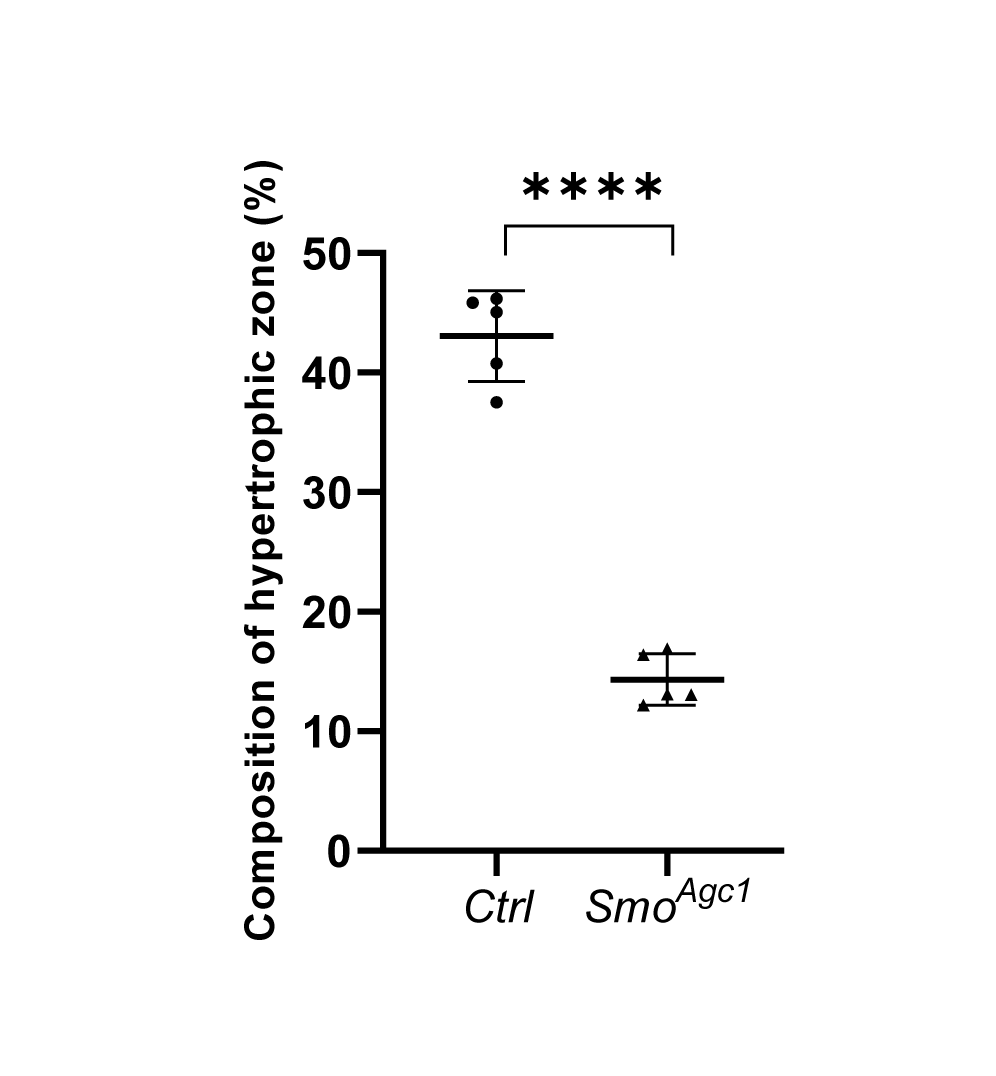

Supplement: Supplementary file 3 [file Image2.TIF]

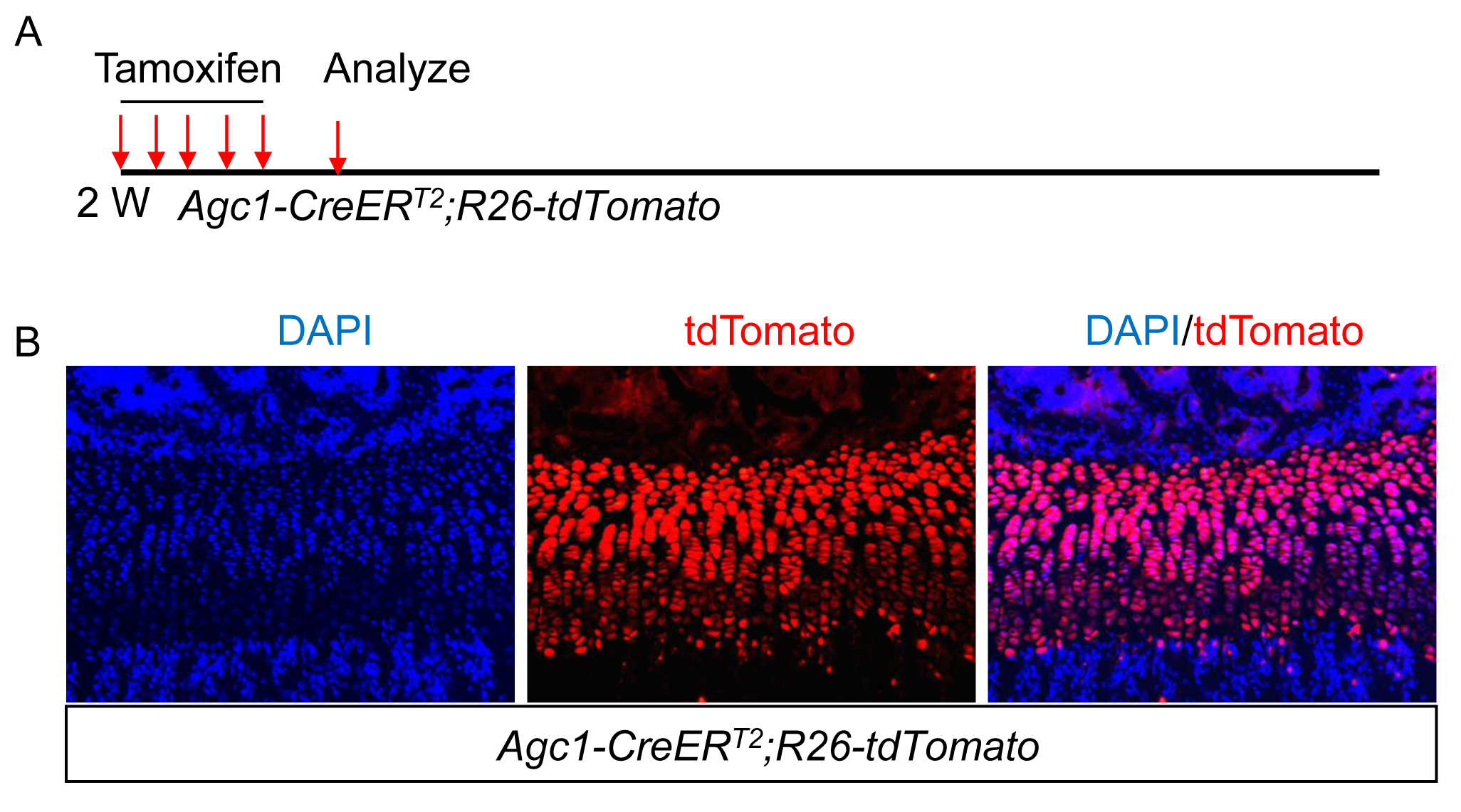

Supplement: Supplementary file 4 [file Image1.TIF]
